# Correlation Between Anti-gp41 Antibodies and Virus Infectivity Decay During Primary HIV-1 Infection

**DOI:** 10.3389/fmicb.2018.01326

**Published:** 2018-06-20

**Authors:** Naveen K. Vaidya, Ruy M. Ribeiro, Pinghuang Liu, Barton F. Haynes, Georgia D. Tomaras, Alan S. Perelson

**Affiliations:** ^1^Department of Mathematics and Statistics, San Diego State University, San Diego, CA, United States; ^2^Theoretical Biology and Biophysics Group, MS K710, Los Alamos National Laboratory, Los Alamos, NM, United States; ^3^Laboratório de Biomatemática, Faculdade de Medicina, Universidade de Lisboa, Lisboa, Portugal; ^4^Harbin Veterinary Research Institute, Chinese Academy of Agricultural Sciences, Harbin, China; ^5^Duke University School of Medicine, Durham, NC, United States

**Keywords:** antibodies, primary HIV-1 infection, viral dynamics model, viral load, virus infectivity

## Abstract

Recent experiments have suggested that the infectivity of simian immunodeficiency virus (SIV) and human immunodeficiency virus type-1 (HIV-1) in plasma decreases over time during primary infection. Because anti-gp41 antibodies are produced early during HIV-1 infection and form antibody-virion complexes, we studied if such early HIV-1 specific antibodies are correlated with the decay in HIV-1 infectivity. Using a viral dynamic model that allows viral infectivity to decay and frequent early viral load data obtained from 6 plasma donors we estimate that HIV-1 infectivity begins to decay after about 2 weeks of infection. The length of this delay is consistent with the time before antibody-virion complexes were detected in the plasma of these donors and is correlated (*p* = 0.023, *r* = 0.87) with the time for antibodies to be first detected in plasma. Importantly, we identify that the rate of infectivity decay is significantly correlated with the rate of increase in plasma anti-gp41 IgG concentration (*p* = 0.046, *r* = 0.82) and the increase in IgM+IgG anti-gp41 concentration (*p* = 8.37 × 10^−4^, *r* = 0.98). Furthermore, we found that the viral load decay after the peak did not have any significant correlation with the rate of anti-gp41 IgM or IgG increase. These results indicate that early anti-gp41 antibodies may cause viral infectivity decay, but may not contribute significantly to controlling post-peak viral load, likely due to insufficient quantity or affinity. Our findings may be helpful to devise strategies, including antibody-based vaccines, to control acute HIV-1 infection.

## Introduction

Primary human immunodeficiency virus type 1 (HIV-1) infection is associated with an initial eclipse phase, during which the viral load remains below the limit of detection of conventional assays, followed by a rapid viral load increase (Daar et al., 1991; Schacker et al., 1996; Fiebig et al., 2003; Ribeiro et al., 2010; Cohen et al., 2011). After the viral load reaches its peak, it declines and reaches a set-point level (i.e., a quasi-steady state). The early events during primary HIV-1 infection not only have particular relevance for vaccine, microbicide and pre/post-exposure prophylaxis (Chun et al., 1998; Pope and Haase, 2003; Shattock and Moore, 2003; Haase, 2005), they are also important in defining the set-point viral load later in infection (Lifson et al., 1997) and the time period over which a successful vaccine needs to induce a protective response prior to establishment of the latent pool of HIV-1 infected CD4^+^ T cells (Wong and Siliciano, 2003; Johnston and Fauci, 2007).

Based on a previous experiment involving simian immunodeficiency virus (SIV) infection of macaques that revealed a difference in infectivity between virus in plasma obtained 7 days after infection and set-point virus (Ma et al., 2009), we introduced an SIV dynamic model with time-dependent viral infectivity (Vaidya et al., 2010). Also, preliminary data comparing the ratio of the 50% tissue culture infectious dose (TCID_50_) with HIV-1 RNA copy number suggests a decrease in virus infectivity over time during primary infection in HIV-1 infected patients, although the magnitude of this effect varies among subjects (Genevieve Fouda and David Montefiori, Duke University School of Medicine, unpublished data). Although the mechanisms responsible for the decay in viral infectivity have not been established, it has been speculated that binding of antibodies to HIV-1 might be in part responsible (Ma et al., 2009). Consistent with this, during early HIV-1 infection it has been shown that anti-gp41 antibodies are produced and form virion-antibody complexes (Tomaras et al., 2008; Liu et al., 2011).

Here we sought to determine whether these early anti-gp41 antibodies influence HIV infectivity by fitting a mathematical model to frequently measured plasma viral loads obtained from 6 plasma donors. The model, which incorporates a time-dependent infectivity rate, fits the acute infection HIV-1 data well. We show the infectivity decay predicted by our model significantly correlates with the anti-gp41 antibody response observed in these plasma donors.

## Materials and methods

### Experimental data

Sequential HIV-1 viral load data from 6 plasma donors was obtained as previously described (Gasper-Smith et al., 2008; Tomaras et al., 2008; Stacey et al., 2009). The study was approved by the Duke Health Institutional Review Board, protocol number Pro00006579. Each individual donated 600–800 ml of plasma which was frozen within 8 h to −20°C or less. The plasma samples were stored up to 2 months then sent in pools to be serologically screened for HIV. Donors who were HIV-1 positive were notified and deferred from subsequent donation. HIV-1 positive samples were aliquoted, and refrozen at −20°C. Aliquoted samples of plasma donors were quantified with the Roche Amplicore HIV-1 RT PCR Ultra assay by Quest Diagnostics (Lyndhurst, NY), with a lower limit of quantification of 50 HIV-1 RNA copies/ml (Tomaras et al., 2008). There was a median of 9 data points per donor with a median of 4 data points before the viral peak. The median peak viral load was 6.0 (range 4.5–6.8) log_10_ viral RNA (vRNA) copies/ml. In these plasma donors, the anti-gp41 IgG and IgM responses were also measured and recorded as optical density (O.D.) (Tomaras et al., 2008). In addition, circulating antibody-virion immune complexes were measured (Tomaras et al., 2008; Liu et al., 2011). The data analyzed below is provided in Table S1.

### Viral dynamic model

To study the effect of antibody responses in decreasing viral infectivity early during infection, we use the standard model of viral infection (Phillips, 1996; Nowak et al., 1997; Little et al., 1999; Perelson and Nelson, 1999; Stafford et al., 2000), but allow the virus infectiousness to decay in time after a certain delay τ, which accounts for the time needed to generate an anti-HIV-1 response. The model is

(1) $$\begin{array}{l} \frac{dT}{dt}=\lambda -dT-\beta \left(t\right)TV,\;T\left(0\right)=T_{0},\\ \frac{dI}{dt}=\beta \left(t\right)TV-\delta I,\;I\left(0\right)=I_{0},\\ \frac{dV}{dt}=pI-cV,\;V\left(0\right)=V_{0}, \end{array}$$

where

(2) $$\beta \left(t\right)=\{\begin{array}{c} \beta_{0},\;t\leq \tau ,\\ \beta_{\infty }+\left(\beta_{0}-\beta_{\infty }\right)e^{-k(t-\tau )},\;t>\tau . \end{array}$$

The model consists of target cells (CD4^+^ T cells), *T*, productively infected CD4^+^ T cells, *I*, and free virus, *V*. We assume that target cells are generated at a constant rate λ, have a per capita net loss rate *d*, which is the difference between loss from cell death and gain due to cell division, and become infected at a rate proportional to the product of target cell density and virus concentration with a time-dependent rate β(*t*). The parameters δ, *p*, and *c* are the rate constants of infected cell loss, virus production by infected cells and virus clearance, respectively. As in Vaidya et al. (2010), we assume a simple exponential decay in infectivity over time from the initial rate β_0_ to the final rate β_∞_ with a decay rate *k*, but for a more general formulation here we include a time-delay τ before infectivity decay begins.

### Data fits and parameter estimation

We fit the model, Equations (1) and (2), to plasma viral load data obtained from 6 HIV-1-infected plasma donors during the acute phase of infection. Earlier studies have shown that the percentage of proliferating CD4^+^ T cells in the peripheral blood of healthy individuals, as measured by Ki-67 antigen expression, is ~1% (Sachsenberg et al., 1998). We use Ki-67^+^ CD4^+^ cells as a surrogate for target cells and thus take the initial number of target cells, *T*_0_, as 10^4^ per ml (1% of 10^6^/ml CD4^+^ T cell count). We note that, as in Stafford et al. (2000), the model system (1) becomes independent of *T*_0_ if the scaling *p*→*p*/*T*_0_ is performed. This shows that taking the value of *T*_0_ different from 10^4^ per ml affects the estimates of only *p*, not the infectivity rate, β(*t*), and thus, our conclusions will remain unaffected if one uses other values of *T*_0_. Assuming CD4^+^ T cells were at equilibrium before infection, we set λ = *dT*_0_. Because the route of infection of the plasma donors is not known, we first assumed infection was initiated by free virus particles rather than infected cells, and thus we set *I*_0_ = 0 (Pearson et al., 2011). Then we also analyzed the data assuming infection was initiated by an infected cell. Recent estimates show that the virion clearance rate constant, *c*, varies between 9.1 day^−1^ and 36.0 day^−1^, with an average of 23 day^−1^ (Ramratnam et al., 1999). Thus, we take *c* = 23 day^−1^, although other values in this range were also considered in a sensitivity analysis.

It is difficult to obtain information about the initial virus concentration that established infection. At least one virion, i.e., 2 viral RNA (vRNA) copies, is needed to establish infection. A 70-kg person has about 15 L of extracellular body water and about 3 L of plasma. Thus, the initial plasma viral load needed to establish systemic infection is >2 vRNA copies per 3,000 ml or >2 vRNA copies per 15,000 ml depending upon whether the virus distributes throughout only the plasma or the total extracellular body water before initiating infection. Here, we present results with *V*_0_ = 10^−3^ vRNA copies per ml assuming that the virus distributes in the plasma and then study the sensitivity of parameter estimates on the initial viral load (*V*_0_) by varying *V*_0_ from 10-fold lower considering the possibility of virus being distributed through extracellular body water to 1,000-fold higher corresponding to the possibility of much higher levels of virus initially entering the circulation.

The exact time of initial infection is not available for this data set. However, the initial viral expansion rates for these subjects have been estimated in a previous study (Ribeiro et al., 2010). Using the slope of viral increase estimated in Ribeiro et al. (2010) and the base value of *V*_0_, we calculated the time of infection and then the time to the first measured viral load above the detection limit for each of these subjects. This allowed us to associate a time since infection with each data point. To estimate τ, we varied τ in 1 day increments, and chose the one which provided the best fit for each plasma donor. The other 6 parameters, β_∞_, β_0_, *k*, δ, *d*, and *p*, were kept free and estimated by fitting the model to the data from each plasma donor. We also performed fitting by making τ a free parameter and obtained approximately the same value as the best estimate from 1-day increment fitting. Since the fit was not improved with τ as an extra free parameter, we fixed τ as the best estimate obtained from the 1-day increment fitting.

Parameter identifiability in HIV models, including those with time-varying parameters, was discussed in Wu et al. (2008) and Miao et al. (2011). As shown in Miao et al. (2011) and Wu et al. (2008), with λ fixed as in our case, all the constant parameters are structurally identifiable. Miao et al. (2011) showed that the time-varying parameter (β(*t*) in our case) is also identifiable if all the constant parameters are identifiable. Therefore, we expect that the parameters of our model are identifiable for the number of data points available in this study.

The data fitting protocol used to estimate parameters was as described previously in Vaidya et al. (2010). We solved the system of ordinary differential equations (ODEs) numerically using a fourth-order Runge-Kutta in Berkeley Madonna. Using Madonna's “curve fitter” option, we obtain a set of initial parameter estimates. The curve fitting method uses nonlinear least-squares regression that minimizes the following sum of the squared residuals:

(3) $$\begin{array}{l} J\left(\beta_{0},\beta_{\infty },k,\delta ,p,d\right)=\frac{1}{N}\overset{N}{\underset{i=1}{\sum }}={\left[\log_{10}\;V(t_{i})-\log_{10}\bar{V}(t_{i})\right]}^{2}. \end{array}$$

Here, *V* and $\bar{V}$ are virus concentrations predicted by the model and those given by the experimental data, respectively. *N* is the total number of data points.

Using the set of parameters obtained from Madonna as initial guesses, we refined the fits by using “fmincon.m” and/or “fminsearch.m” functions in MATLAB. For each best fit parameter estimate, we provide a 95% confidence interval (CI), which was computed from 500 bootstrap replicates (Efron and Toibshirani, 1986). Since we analyze only 6 subjects, we present results as medians and ranges, unless otherwise indicated.

### Sensitivity analysis

The viral load establishing systemic infection, *V*_0_, is not known. To study the sensitivity of our results to the choice of *V*_0_, we randomly selected 200 different *V*_0_ from 10-fold lower (i.e., 10^−4^ vRNA copies/ml) to 1,000-fold higher (i.e., 1 vRNA copies/ml) and estimated parameters for each of the 6 donors.

### Statistical analysis

We performed linear regression to obtain the slope of the IgG increase, the IgM increase and the IgG+IgM increase. We then carried out correlation analyses using Pearson's correlation between these slopes and the decay slope of infectivity estimated by our model. We also calculated the slope of the viral load decay after the peak and performed correlation analyses of the viral decay rate with the antibody response.

To evaluate the statistical significance of models comparisons, we performed an *F*-test (Bates and Watts, 2007) as the models considered in this study without and with infectivity decay are nested.

## Results

### Model fitting to data

**Figure 1:**
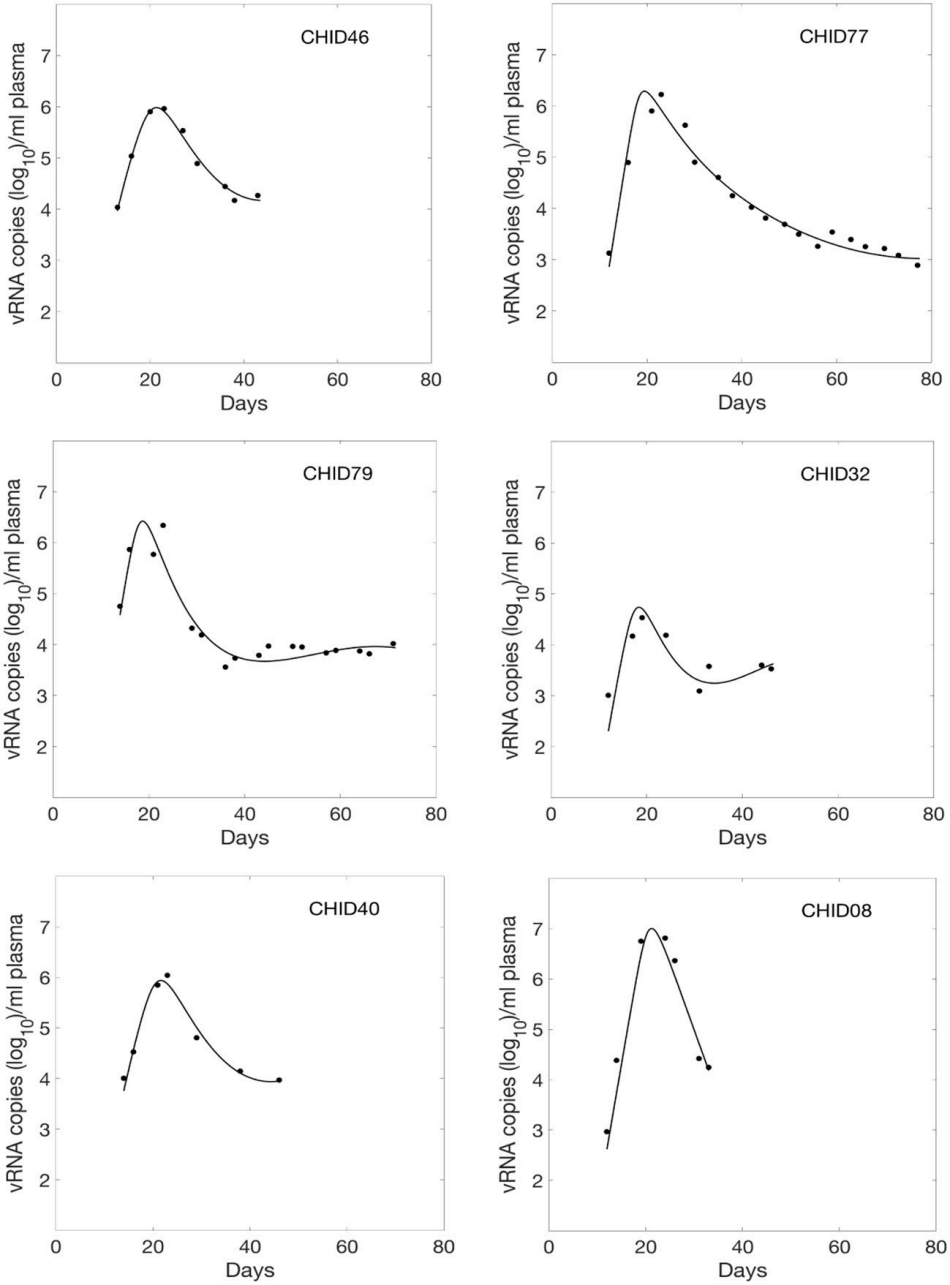
Fitted viral dynamics curve using the delay model with time-varying infectivity to the observed viral load data (filled circle) during primary infection of 6 HIV-1 infected plasma donors.

We fitted Equations (1) and (2) to the HIV-1 data. We estimated six parameters β_∞_, β_0_, *k*, δ, *d*, and *p* from the data fitting. The estimated parameters along with their 95% confidence intervals are summarized in Table 1. Using these estimated parameters, we plotted the viral load dynamics predicted by the model along with the data for each of the 6 HIV-1 infected plasma donors in Figure 1. The predictions of our time-varying infectivity delay model (solid curve) agree well with the data (filled circles).

**Table 1:** Estimated parameter values β_0_, β_∞_, *k*, δ, *p, d*, τ, and time *t*_*h*_ to reach the mid-value (β_0_ + β_∞_)/2.

**Patient**	**β_0_ (10^−6^ml/RNA/day)**	**β_∞_ (10^−6^ml/RNA/day)**	***k* (1/day)**	**δ (1/day)**	***p* (10^3^ RNAs/day)**	***d* (1/day)**	**τ (day)**	***t_*h*_*(day)**
CHID46	0.409 (0.376–0.441)	0.233 (0.169–0.297)	0.249 (0.234–0.250)	0.775 (0.737–0.814)	14.500 (14.499–14.501)	0.030 (0.023–0.038)	7	2.8
CHID77	0.431 (0.417–0.444)	0.140 (0.129–0.151)	0.077 (0.067–0.093)	0.420 (0.417–0.433)	10.000 (9.999–10.001)	0.021 (0.019–0.025)	24	9.0
CHID79	0.201 (0.195–0.208)	0.001 (0.000–0.027)	0.013 (0.012–0.015)	1.048 (0.992–1.064)	30.172 (30.166–30.178)	0.036 (0.033–0.041)	10	53.3
CHID32	9.203 (4.320–11.011)	0.011 (0.000–0.112)	0.013 (0.011–0.020)	0.851 (0.325–1.360)	0.548 (0.391–0.901)	0.055 (0.048–0.156)	12	53.3
CHID40	0.485 (0.457–0.513)	0.291 (0.161–0.339)	0.096 (0.062–0.103)	0.803 (0.623–0.910)	11.425 (11.422–11.428)	0.033 (0.022–0.037)	5	7.2
CHID08	0.057 (0.050–0.112)	0.004 (0.000–0.019)	0.021 (0.019–0.031)	0.821 (0.491–1.170)	89.892 (48.541–130.952)	0.003 (0.000–0.028)	22	33.0
Median	0.420	0.076	0.049	0.812	12.962	0.032	11	21.0

*Numbers in parentheses indicate 95% confidence intervals (see Materials and Methods)*.

For comparison, we also fitted these viral load data using a constant infectivity (i.e., β(*t*) constant) model (Stafford et al., 2000), and found that the delay model with time-dependent infectivity provides statistically significant better fits (*p* = 0.001, *F*-test with all the subjects combined as in Vaidya et al., 2010). Moreover, we compared the data fitting using a time-dependent model without delay (Vaidya et al., 2010) (i.e., τ = 0), and found that including a delay in the model significantly improved the fits (*p* = 0.008, *F*-test, Vaidya et al., 2010).

### Virus infectivity decay

We estimated the median initial and late viral infection rate constants to be β_0_ = 4.20 × 10^−7^ ml RNA^−1^ day^−1^ and β_∞_ = 0.76 × 10^−7^ ml RNA^−1^ day^−1^, respectively (Table 1). This suggests that infectivity decays during acute HIV-1 infection (*p* = 0.031, paired Wilcoxon Test). Such infectivity decay over time was also observed previously in SIV infection (Ma et al., 2009; Vaidya et al., 2010). Assuming that the decay of β(*t*) occurs exponentially with rate *k*, we found that HIV-1 infectivity decays with a median rate of *k* = 0.049 day^−1^ (Table 1) (range: *k* = 0.013 day^−1^ to *k* = 0.249 day^−1^). Also, the time, *t*_*h*_, to reduce the virus infectivity to its mid-value, (β_0_ + β_∞_)/2, given by ln(2)/*k*, was found to be 21 days (Table 1).

### Correlation of infectivity with antibody response

It is known that antibodies bind to virions and form antibody-virion complexes (Dianzani et al., 2002; Tomaras et al., 2008; Liu et al., 2011). Such antibodies bound to virions might interfere with the infection process (Ma et al., 2009). Therefore, we examined if there is any correlation between the infectivity decay and the earliest antibody responses detected during acute infection, i.e., the anti-gp41 IgM and/or IgG response (Tomaras et al., 2008; Liu et al., 2011).

**Figure 2:**
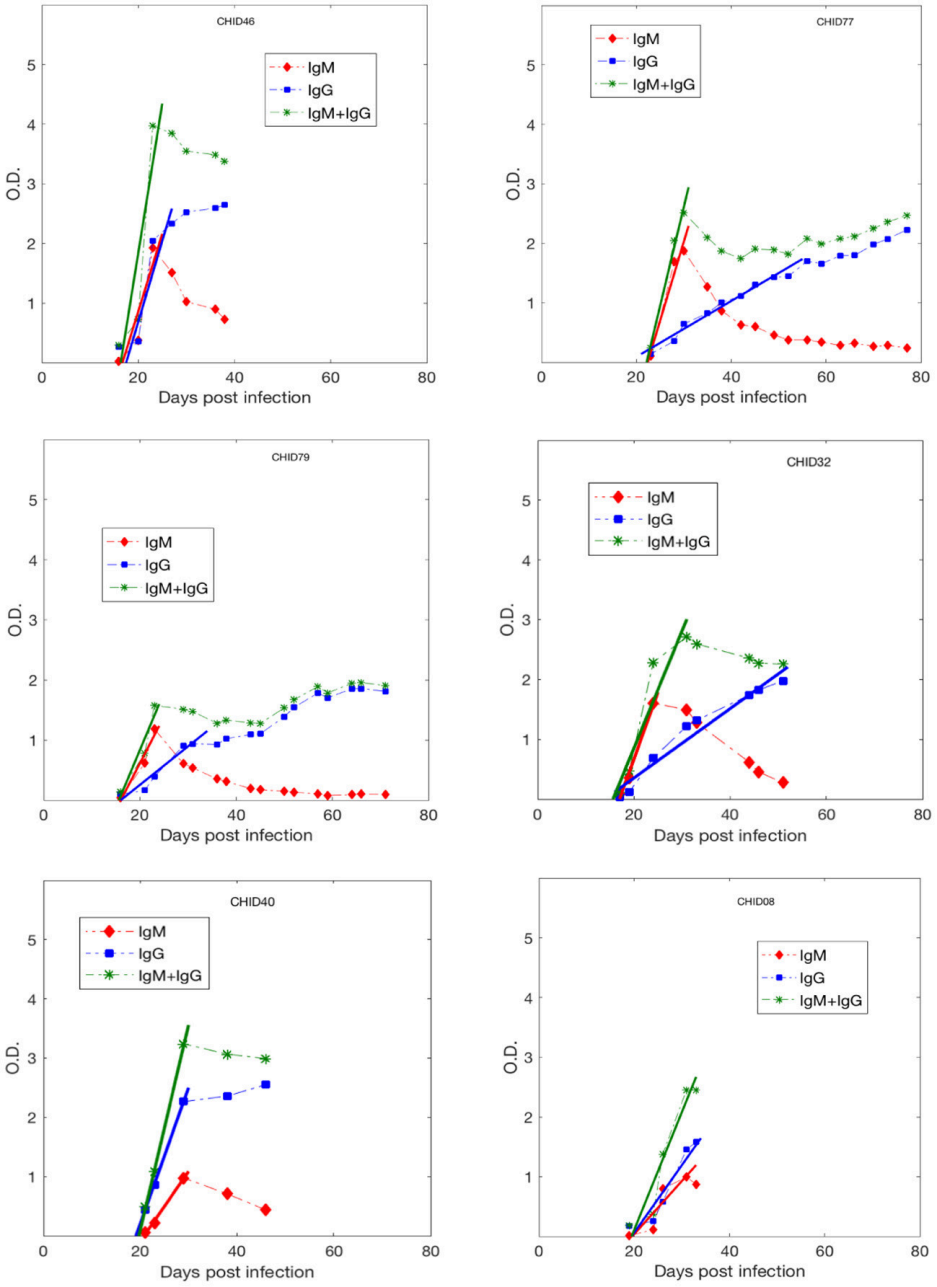
Anti-gp41 IgM, IgG and (IgM+IgG) antibody response data during primary infection from 6 HIV-1 infected plasma donors. The lines represent the best fits used to estimate the upward slope of the antibody increase.

While we acknowledge some uncertainty due to sparsity in early Ab data, in general, as shown in Figure 2, the anti-gp41 IgM concentration (measured in optical density. i.e., O.D. units) increases approximately linearly up to a maximum value and then decays, whereas the anti-gp41 IgG concentration increases monotonically over the time period studied. This pattern of IgM increasing and then decreasing is consistent with the known features of the IgM-IgG isotype switch (Murphy et al., 2008). We performed a linear regression analysis to find the slope of the IgM increase, of the IgG increase and of the IgM+IgG increase using the antibody data to the time point where antibody levels saturate or start to decay. The IgM and IgG concentrations increase by a median rate of 0.19 day^−1^ and 0.09 day^−1^, respectively, while the median rate of increase in the IgM + IgG concentration is 0.27 day^−1^ (Table S2).

**Figure 3:**
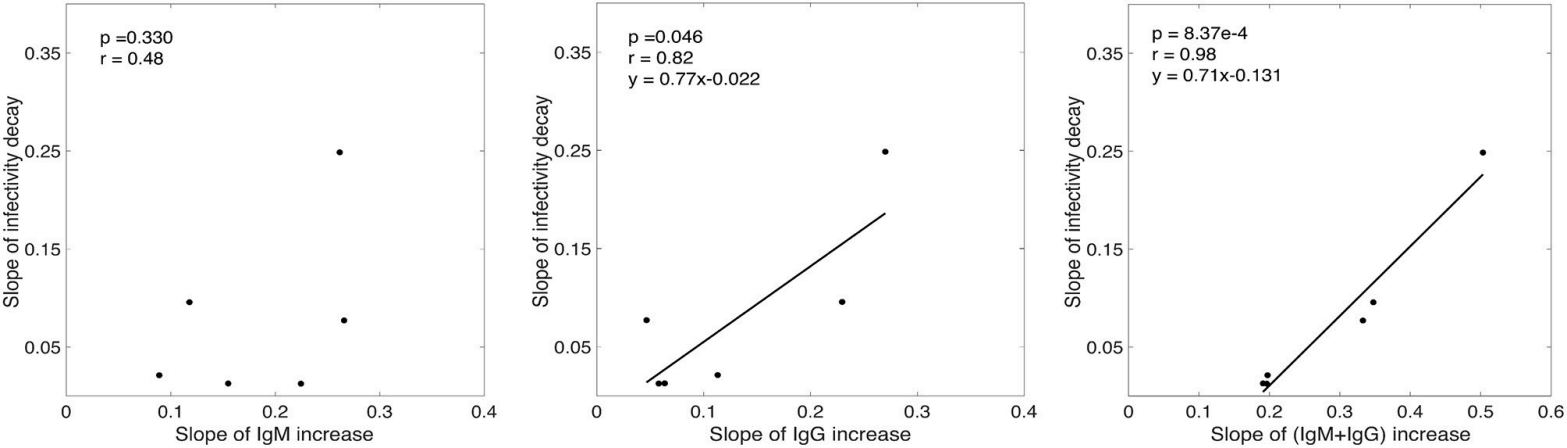
Correlation analysis of the slope of experimentally measured IgM, IgG and (IgM+IgG) antibody increase with the rate of infectivity decay predicted by our model.

While there was a positive association between the rate of infectivity decay estimated by our model (*k*) and the slope of IgM increase (Figure 3), this correlation was not statistically significant (*p* = 0.33, *r* = 0.48). However, we found that the rate of infectivity decay has a statistically significant positive correlation with the slope of IgG increase (*p* = 0.046, *r* = 0.82) and a very significant positive correlation with the IgM+IgG anti-gp41 concentration with *p*-value = 8.37 × 10^−4^ and *r*-value = 0.98 (Figure 3). This suggests that the antibody response might contribute to the loss of virus infectivity. To check the robustness of this finding, we performed correlation analysis by iteratively excluding each donor one at a time, and found that the correlation of infectivity decay with slope of increase of IgM+IgG remained statistically significant (*p* < 0.01 in each case, Table S3).

### The delay before the start of infectivity decay correlates with the time until the antibody response is detected

**Figure 4:**
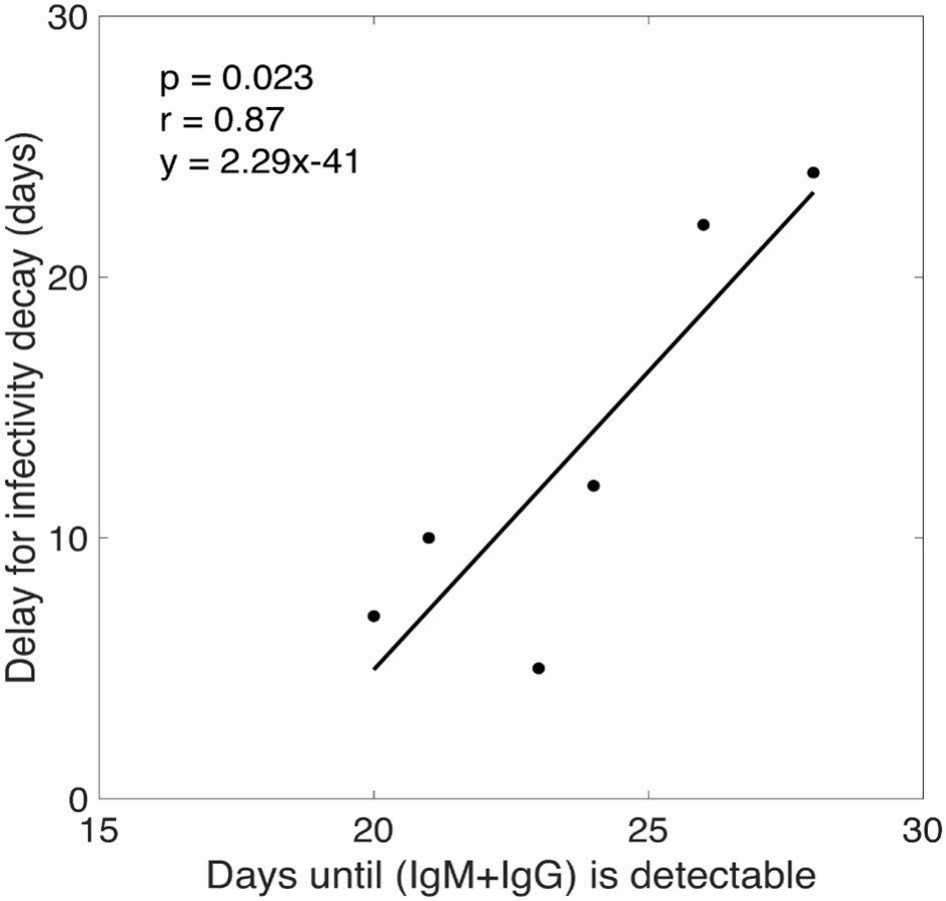
Correlation analysis between the time for total antibody (IgM+IgG) response to be experimentally detectable in plasma and the delay for the start of infectivity decay predicted by our model.

Our model predicts that the virus infectivity begins to decay after a median time of 11 days (range: 5–24 days) of infection. The exact delay from the time of infection to the initiation of antibody increase is not known. However, from the experimental data we estimated the time from infection (as estimated by our calculation) to the time when the free IgM+IgG level begin to increase in plasma. In the donated plasma, antibodies were measured and, in every case, O.D. readings of both IgM and IgG began to increase on the same day. Since the antibodies were assayed in every sample, we defined the time when antibody becomes detectable as the first time point for which the O.D. of IgM+IgG level was above the limit of detection (i.e., O.D. >0.5). We found a statistically significant correlation (*p* = 0.0233, *r* = 0.87) between the time that antibody became detectable in plasma and the delay before infectivity decay began predicted by our model (Figure 4). Furthermore, for three donors (CHID77, CHID08, CHID79), the times for antibody-virion complexes to be experimentally detectable in plasma were reported previously as 13, 9, and 6 days, respectively, where this was measured relative to the time at which the plasma viral load first reached 100 copies/ml (Tomaras et al., 2008). Using the eclipse phase of acute infection in these plasma donors, calculated from the slope of viral increase estimated in Ribeiro et al. (2010), these times translate to 24, 18, and 14 days from the time of infection. These values and their rank-order are consistent with the delay for infectivity decay predicted by our model (24, 22, 10 days, respectively, Table 1).

### Correlation of post-peak viral load drop with antibody response

To observe if antibodies have any significant impact on viral load decay after the viral load peak, we performed a correlation analysis between the slope of IgM increase, IgG increase, IgM+IgG increase and the slope of the viral load drop after the peak (Table S2). We did not find any significant correlation with IgM, IgG or IgM+IgG indicating that this antibody response might not be the primary cause for the drop of viral load after the peak, consistent with previous findings (Tomaras et al., 2008). In our viral dynamic model, Equation (1), viral load drop after the peak is due to target cell limitation and death of productively infected cells.

### Sensitivity analysis

Above we analyzed the correlation of two parameters, *k* and τ, with the antibody response. We estimated these parameters by fitting our model to viral load data. Due to lack of information about the actual number of virions initiating infection, *V*_0_, we assumed *V*_0_ = 10^−3^ vRNA copies/ml. To ensure that the choice of *V*_0_ did not bias our results, we re-fit the data taking 200 different values of *V*_0_ selected randomly from 10-fold lower to 1,000-fold higher (i.e., 10^−4^ to 1) than the base-case. We find that the estimate of τ is not affected at all, and that the median change in the estimates of *k* is below 5% (Figure S1). Therefore, our results are not sensitive to the choice of *V*_0_.

We assumed that the infection was initiated with free virus particles. To study how the estimates are affected if the infection was initiated with infected cells, we compared the estimates between an infection with one virus particle distributed in 15 L body water (i.e., *V*_0_ = 2/15000 vRNA copies/ml) and an infection with one infected cell distributed in 15 L body water (i.e., *I*_0_ = 1/15000 cells/ml). We found that the estimates of *k* are essentially the same in these two cases (Figure S2).

We chose *c* = 23 d^−1^ based on the average of the experimentally estimated range between 9 and 36 d^−1^. To test the robustness of our results to this assumption, we refitted the data with different values of c within this range. The only parameters that is mainly affected is the viral production rate. Therefore, our results regarding *k* and τ are not affected by the specific value of *c*.

## Discussion

During primary HIV-1 infection, a decay of virus infectivity over time has been suggested by comparing the ratio of tissue culture infectious dose (TCID_50_) with HIV RNA copy number in sequential early viral load samples from a limited number of subjects (Genevieve Fouda and David Montefiori, unpublished data). In addition, HIV-1-specific anti-gp41 antibodies have been detected in plasma a median of 13 days after the viral load reaches 100 vRNA copies/ml (Tomaras et al., 2008). Moreover, anti-gp41 IgM-virion or IgG-virion complexes were found as early as 5 days after the viral load became detectable (Tomaras et al., 2008; Liu et al., 2011). The presence of such antibodies might affect the infectivity of HIV-1 (Tomaras et al., 2008; Ma et al., 2009). Therefore, one of the main objectives of this study was to ask if there is a correlation between the infectivity decay of plasma virus and the anti-gp41 antibody response in HIV-1 infected individuals.

Since there are delays before antibodies and antibody-virion complexes become detectable in plasma (Tomaras et al., 2008), we extended a previous infection model (Vaidya et al., 2010) used to study acute SIV infection by incorporating a time-delay before infectivity decay begins. We then used this delay model to quantify the time-variation of HIV-1 infectivity during primary infection. Our data fitting procedure reveals that both time-dependent nature and delay of infectivity decay are necessary to better describe the viral load data from primary HIV-1 infection.

According to our model estimates, plasma HIV-1 infectivity decays exponentially with a median rate of 0.049 day^−1^ (Table 1), and there is a time delay of about 2 weeks (range 5–24 days) before virus infectivity begins to decay. The length of this delay is consistent with the period from infection to the time when the virion-antibody complexes were detected in plasma (Tomaras et al., 2008), and is significantly correlated (*p* = 0.0233, *r* = 0.87) with the time post-infection for anti-gp41 antibody (IgG+IgM) to be detectable in plasma (Figure 4).

Our analyses also showed a statistically significant and strong correlation between the rate of increase of the IgM+IgG anti-gp41 antibody concentration and the rate of infectivity decay estimated by the model (*p* = 0.0008, *r* = 0.98) (Figure 3). On the other hand, we did not observe a significant correlation between the slope of the IgM, IgG or IgM+IgG increase and the slope of viral load drop after the viral load peak. Taken together, these results indicate that the anti-gp41 (IgM+IgG) response might contribute to the reduction of virus infectivity, but that these anti gp41 antibodies have minimal effect on controlling post peak viral load as seen in Tomaras et al. (2008). Thus other factors, such as target cell limitation (Stafford et al., 2000) and cytotoxic T cell responses (Goonetilleke et al., 2009) may be playing a role in determining the post-peak viral decline. Because cells are not collected from plasma donors quantifying the change in target cell levels and the magnitude of the CTL response was not possible in this study.

A contribution of antibodies to reducing viral infectivity was suggested by Ma et al. (2009), and supported by their observation that mixing plasma obtained at set-point with plasma obtained 7 days after SIV infection reduced the infectivity of the 7-day plasma. However, our inference that antibody affects the infectivity of HIV-1 during early infection is derived from a correlation based on limited viral load and antibody data from only 6 individuals. We cannot rule out other possible causes of infectivity decay such as the production of non-infectious viral genomes that reduce infectivity, as the virus that founds the infection diversifies due to mutation during early infection, or other plasma proteins binding to virions and mediating infectivity decay. Also, the correlation between the slope of the infectivity decay and the up-slope of antibody responses obtained in this study is for the early stages post-infection. Once a plasma donor was identified as being HIV+ donations were stopped and hence no long-term data were collected. Later in the infection antibody responses saturate or decay. To capture the long-term effect, the model needs to be extended to incorporate such behavior and longer-term data is needed to validate such extended models.

While this study supports the hypothesis that antibodies reduce viral infectivity, we acknowledge that antibodies might have other anti-HIV effects, such as enhanced virion clearance and/or antibody-dependent cellular cytotoxicity (Tomaras and Haynes, 2009, 2010). However, these effects were found to have negligible contribution to HIV-1 viral dynamics (Tomaras et al., 2008). In our previous study, we (Tomaras et al., 2008) also investigated the effects of antibody in neutralizing virus by reducing the infectivity rate in a mathematical model including antibody data, but we did not find a significant antibody effect in most patients. The difference with the current results could be due to differences in the two modeling approaches: the delay in the antibody effect in Tomaras et al. (2008) was entirely given by the free antibody data, i.e., the delay corresponded to the time delay for antibody to become detectable in plasma, while the delay in our model (estimated to be much shorter, Figure 4) corresponds to the delay for the formation of antibody-virion complexes. Note that antibody-virion complexes are detectable earlier than free antibodies in plasma (Tomaras et al., 2008). The second difference in the two modeling approaches is the functional form of the infectivity decay introduced into the models (see Text S1). A study with more antibody data may help to accurately and explicitly incorporate antibody effects into viral dynamic models. While direct comparison between these two models might not be appropriate as our model does not have explicit dynamics for antibodies, clarifying these issues might be important for future development of models that take explicit antibody responses into account. We also acknowledge uncertainty in the route of infection and the actual time of infection; if the time of initial infection is different, then this may imply a different dose of infecting virus, or even differences in host immune response to the virus infection. However, we note that it is very difficult to find HIV infected individuals so early in infection. This complexity makes this data set unique and highlights the importance of this study.

Although our model cannot conclusively address the causes of decay in HIV-1 infectivity, the quantitative agreement between our model's predictions and the measured viral load curves in all 6 subjects, and the correlation of the rate of infectivity decay with the measured increase in anti-gp41 antibody concentrations strongly suggest the early anti-HIV-1 response, even though non-neutralizing may still provide benefit. More data, especially on early antibody responses (including IgA responses), the formation of antibody-virion complexes, and the ratio of infectious virus to total HIV-1 RNA are needed to provide a more accurate picture of virus infectivity during primary HIV-1 infection.

## Author contributions

NV and AP designed the study. NV performed mathematical analysis and numerical experiments. NV, RR, and AP analyzed the data. PL, BH, and GT provided the experimental data. All authors contributed to writing the paper.

### Conflict of interest statement

The authors declare that the research was conducted in the absence of any commercial or financial relationships that could be construed as a potential conflict of interest. The reviewer SW and handling Editor declared their shared affiliation.
