# Supplementary material for: Correlation Between Anti-gp41 Antibodies and Virus Infectivity Decay During Primary HIV-1 Infection
Source: Front Microbiol. 2018 Jun 20;9:1326. doi: 10.3389/fmicb.2018.01326 (PMC6019451; doi:10.3389/fmicb.2018.01326)
Supplement: Supplementary file 1 [file Presentation_1.PDF]

Supplementary Materials

Figure S1. Sensitivity analysis of the choice of V_0. Percentage change in the estimates of the slope of infectivity decay, k, obtained for 200 different values of V_0 selected randomly from 10^{-4} to 1 vRNA copies/ml.

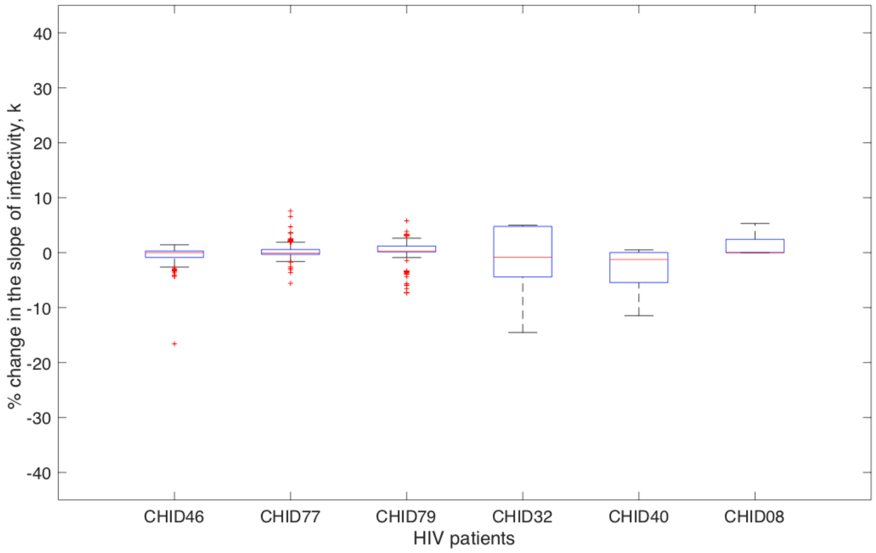

Figure S2. Infection initiated by a free virus particle vs. by an infected cell. Estimates of the slope of infectivity decay, k, when the infection is initiated with one virus particle or with one infected cell.

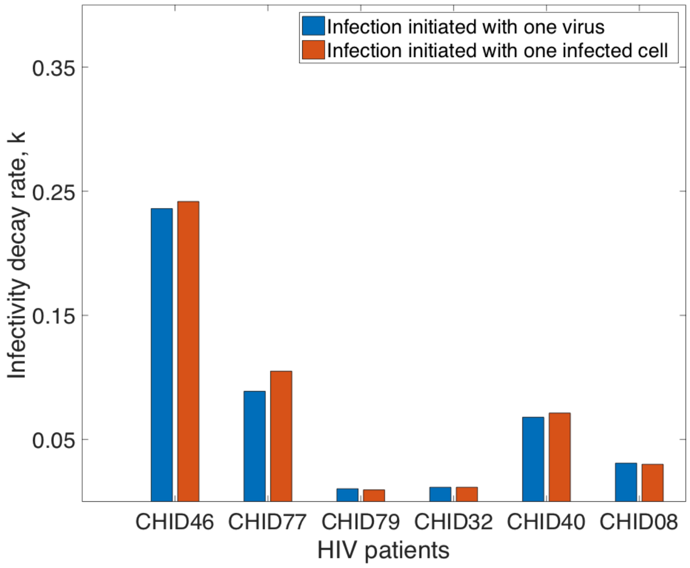

Text S1. Difference in the infection rate decay between our model and the Tomaras et al. (2008) model.

The infection rate in our model is given by

$$\beta(t) = \begin{cases} \beta_0, & t \le \tau_1, \\ \beta_0 + (\beta_0 - \beta_\infty) e^{-k(t-\tau_1)}, & t > \tau_1. \end{cases} \qquad (1)$$

For $t > \tau_1$, the rate of change of $\beta(t)$ per unit time is

$$\frac{d\beta(t)}{dt} = -k(\beta_0 - \beta_\infty) e^{-k(t-\tau_1)}.$$

From (1), $(\beta_0 - \beta_\infty) e^{-k(t-\tau_1)} = \beta(t) - \beta_0$. Therefore,

$$\frac{d\beta(t)}{dt} = -k[\beta(t) - \beta_0].$$

Tomaras et al. (2008) introduces the effect of antibodies directly into the viral dynamics model using the following function for the rate of infection:

$$\beta(t) = \frac{\beta_0}{1 + \alpha Ig(t)}$$

where $Ig(t)$ is the functional curve representing either the concentration of anti-gp41 IgM or anti-gp41 IgG or the total of both Ig concentrations in plasma, and α is scaling factor. The expression for $Ig(t)$ curve was obtained from a spline curve fit to the measured data. Therefore, in the Tomaras et al. model, the delay in infectivity decay, τ_2, is imposed by the delay of Ig concentrations observed in the data, and after τ_2, the rate of change of $\beta(t)$ per unit time is

$$\frac{d\beta(t)}{dt} = -\alpha\beta_0 \frac{dIg(t)}{dt} \frac{1}{[1 + \alpha Ig(t)]^2} = -\left[\frac{\alpha}{\beta_0} \frac{dIg(t)}{dt}\right] \beta(t)^2.$$

Therefore, there are two major differences between our modeling approach and that of Tomaras et al.:

1. The magnitude of the delay before infectivity decays, τ_1 in our model and τ_2 in the Tomaras et al. model. As observed in our calculations, these two values are quite different.
2. The decay rate of infectivity introduced into the models are quite different. In particular, since as shown in Fig. 2 the antibody concentration initially expands linearly (such that dIg/dt is a constant), the decrease in infectivity ($d\beta/dt$) in the Tomaras et al. model is proportional to a quadratic term, whereas in our model it is proportional to a linear term.

Table S1. Experimental data.

Patient	Days post Infection	vRNA copies/ml	IgM (O.D.)	IgG (O.D.)
CHID46	13	10910	-	-
	16	108100	0.026	0.268
	20	800000	0.371	0.354
	23	922270	1.927	2.047
	27	340400	1.512	2.335
	30	77800	1.023	2.526
	36	27540	0.896	2.592
	38	14700	0.728	2.647
	43	18340	-	-
CHID77	12	1353	-	-
	16	79235	-	-
	21	798590	-	-
	23	1680660	0.101	0.136
	28	417325	1.689	0.358
	30	79536	1.875	0.642
	35	40369	1.271	0.828
	38	17838	0.866	1.002
	42	10642	0.629	1.113
	45	6515	0.597	1.308
	49	4935	0.458	1.428
	52	3161	0.376	1.444
	56	1827	0.376	1.701
	59	3474	0.335	1.656
	63	2487	0.286	1.792
	66	1790	0.321	1.797
	70	1646	0.267	1.982
	73	1220	0.285	2.075
	77	777	0.239	2.227
CHID79	14	56508	-	-
	16	731600	0.037	0.100
	21	584913	0.618	0.164
	23	2170000	1.186	0.390
	29	20988	0.610	0.905
	31	15515	0.537	0.937
	36	3594	0.353	0.927
	38	5408	0.307	1.024
	43	6105	0.195	1.093
	45	9353	0.173	1.105
	50	9245	0.148	1.387
	52	8960	0.127	1.544
	57	6832	0.108	1.782
	59	7707	0.080	1.704
	64	7510	0.091	1.852
	66	6554	0.100	1.856
	77	10479	0.097	1.810
CHID32	12	1013	-	-
	17	14737	0.076	0.027
	19	34213	0.352	0.114
	24	25434	1.604	0.683
	31	1238	1.491	1.221
	33	3768	1.280	1.316
	44	4002	0.615	1.740
	46	3372	0.450	1.825
CHID40	14	10081	-	-
	16	33612	-	-
	21	706570	0.055	0.437
	23	1097490	0.217	0.862
	29	63661	0.977	2.264
	38	14084	0.707	2.359
	46	9353	0.436	2.554
CHID08	12	925	-	-
	14	24194	-	-
	19	5631550	0.014	0.175
	24	6486240	0.114	0.258
	26	2296060	0.795	0.585
	31	26311	0.997	1.456
	33	17425	0.872	1.579

Table S2. Slopes of antibody increase and post-peak viral decay (per day).

Patient	IgM	IgG	IgM+IgG	Viral decay
CHID46	0.2616	0.2700	0.5031	0.0926
CHID77	0.2658	0.0465	0.3327	0.0555
CHID79	0.1549	0.0635	0.1908	0.1720
CHID32	0.2245	0.0579	0.1961	0.0895
CHID40	0.1178	0.2296	0.3475	0.0857
CHID08	0.0889	0.1131	0.1972	0.3098

Table S3. Correlation analysis results. P-values and r-values obtained from the correlation analysis between the infectivity decay slope, k, and the slope of antibody (IgM, IgG, and IgM+IgG) increase when each patient was excluded from the analysis. Note that the correlations with IgM+IgG are always stronger than with IgM or IgG individually. One possible biological explanation for the observation is that during the antibody response B cells secreting antibody undergo a class switch from IgM to IgG, while maintaining specificity for the viral epitopes. Thus, it could be that neither IgM nor IgG by itself is enough to correlate with the effect of the response (here measured by k), since B-cells producing IgM are being transformed into B-cells producing IgG. However, when we put the two together, representing the overall strength of the response, there is such a correlation

Patient left out	IgM		IgG		IgM+IgG	
	p-value	r-value	p-value	r-value	p-value	r-value
None	0.3296	0.48	0.0469	0.82	0.0008	0.98
CHID46	0.8819	0.09	0.2921	0.59	0.0008	0.99
CHID77	0.3152	0.57	0.0368	0.90	0.0015	0.99
CHID79	0.4443	0.45	0.1095	0.79	0.0049	0.97
CHID32	0.2508	0.63	0.1114	0.79	0.0052	0.97
CHID40	0.2959	0.59	0.0397	0.89	0.0028	0.98
CHID08	0.5108	0.39	0.0790	0.83	0.0042	0.98
